# Supplementary material for: Persistent immunogenicity of integrase defective lentiviral vectors delivering membrane-tethered native-like HIV-1 envelope trimers
Source: NPJ Vaccines. 2022 Apr 21;7:44. doi: 10.1038/s41541-022-00465-1 (PMC9023570; doi:10.1038/s41541-022-00465-1)
Supplement: Supplementary file 1 — Supplemental Material [file 41541_2022_465_MOESM1_ESM.pdf]

SUPPLEMENTARY INFORMATION FOR THE MANUSCRIPT

Persistent Immunogenicity of Integrase Defective Lentiviral Vectors delivering membrane tethered Native-Like HIV-1 Envelope Trimers

Alessandra Gallinaro[1], Maria Franca Pirillo[1], Yoann Aldon[2,12], Serena Cecchetti[3], Zuleika Michelini[1], Antonella Tinari[4], Martina Borghi[5], Andrea Canitano[1], Paul F. McKay[2], Roberta Bona[1], Maria Fenicia Vescio[5], Felicia Grasso[5], Maria Blasi[6,7], Silvia Baroncelli[1], Gabriella Scarlatti[8], Celia LaBranche[9], David Montefiori[9], Mary E. Klotman[6], Rogier W. Sanders[10,11], Robin J. Shattock[2], Donatella Negri[5], Andrea Cara[1]*

[1]National Center for Global Health, Istituto Superiore di Sanità, Rome, Italy. [2]Imperial College London, Department of Infectious Disease, Norfolk Place, London, UK. [3]Confocal Microscopy Unit NMR, Confocal Microscopy Area Core Facilities, Istituto Superiore di Sanità, Rome, Italy. [4]Center for Gender Medicine, Istituto Superiore di Sanità, Rome, Italy. [5]Department of Infectious Diseases, Istituto Superiore di Sanità, Rome, Italy. [6]Department of Medicine, Division of Infectious Diseases, Duke University School of Medicine, Durham, NC, USA. [7]Duke Human Vaccine Institute, Duke University School of Medicine, Durham, NC, USA. [8]Viral Evolution and Transmission Unit, IRCCS Ospedale San Raffaele, 20132 Milan, Italy. [9]Department of Surgery, Duke University School of Medicine, Durham, NC, USA. [10]Amsterdam University Medical Centers, Amsterdam Institute for Infection and Immunity, University of Amsterdam, Amsterdam, the Netherlands. [11]Department of Microbiology and Immunology, Weill Medical College of Cornell University, 1300 York Avenue, New York, NY, USA. [12]Present address: Amsterdam University Medical Centers, Amsterdam Institute for Infection and Immunity, University of Amsterdam, Amsterdam, the Netherlands.

*Correspondence to: Andrea Cara, National Center for Global Health, Istituto Superiore di Sanità, Viale Regina Elena 299, 00161 Rome, Italy. E-mail: andrea.cara@iss.it

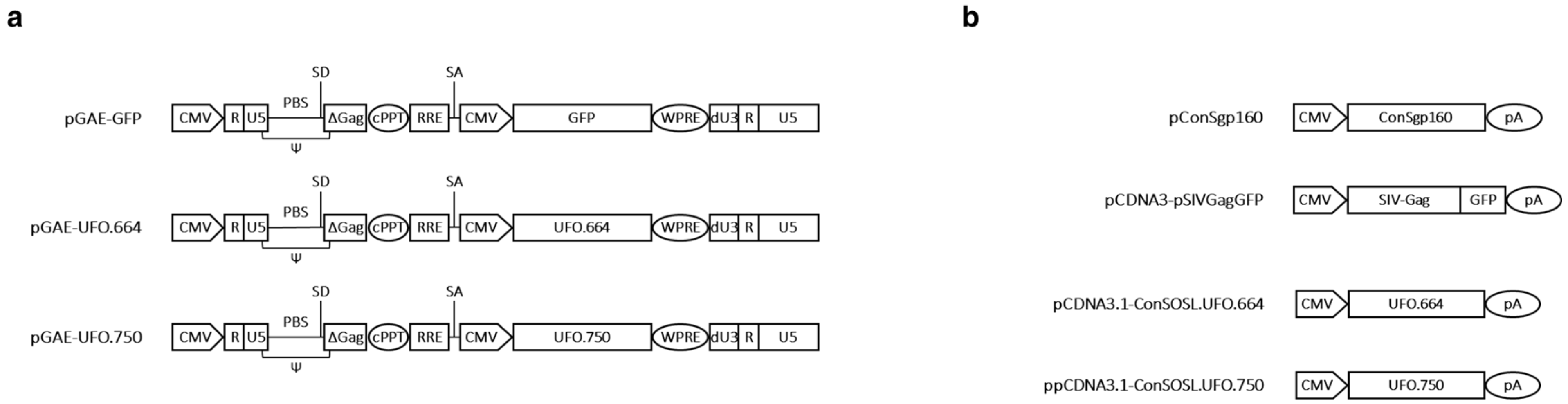

Supplementary Figure 1. Schematic representation of plasmids used in this study. (**a**) SIV-based lentiviral transfer vectors expressing GFP, ConSOSL.UFO.664 or ConSOSL.UFO.750. (**b**) pcDNA3 plasmids expressing ConSgp160, SIV-Gag fused to GFP, ConSOSL.UFO.664 or ConSOSL.UFO.750. CMV, cytomegalovirus immediate-early promoter; R, repeat element; U5, 5' untranslated region; U3, 3' untranslated region; PBS, primer binding site; SD, splice donor site; Ψ, packaging signal; cPPT, central polypurine tract; RRE, Rev response element; SA, splice acceptor site; dU3, SIN deletion in U3 region of 3' LTR; WPRE, woodchuck hepatitis virus post-transcriptional regulatory element. See Methods for details on construction.

Supplementary Figure 2a

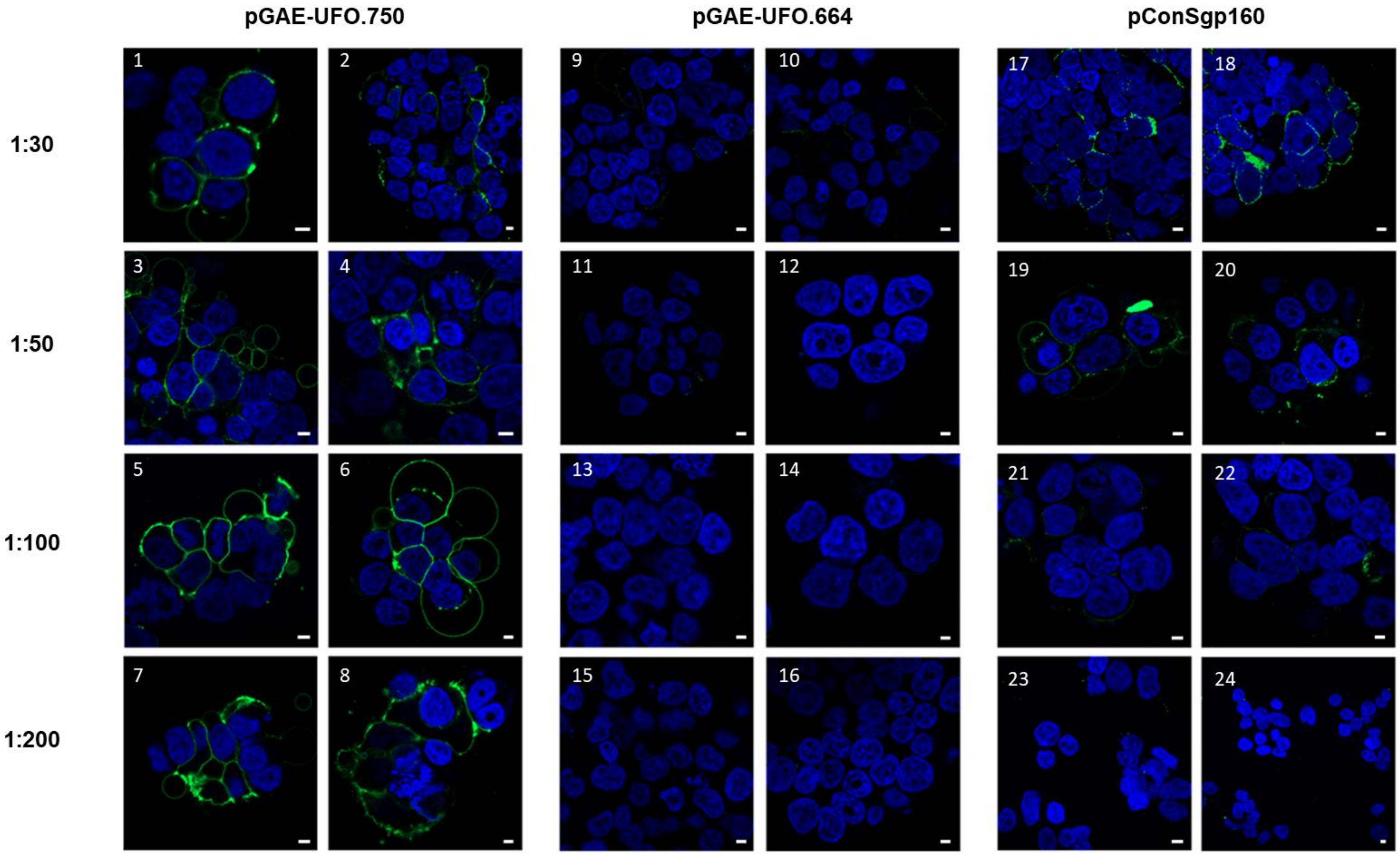

Supplementary Figure 2b

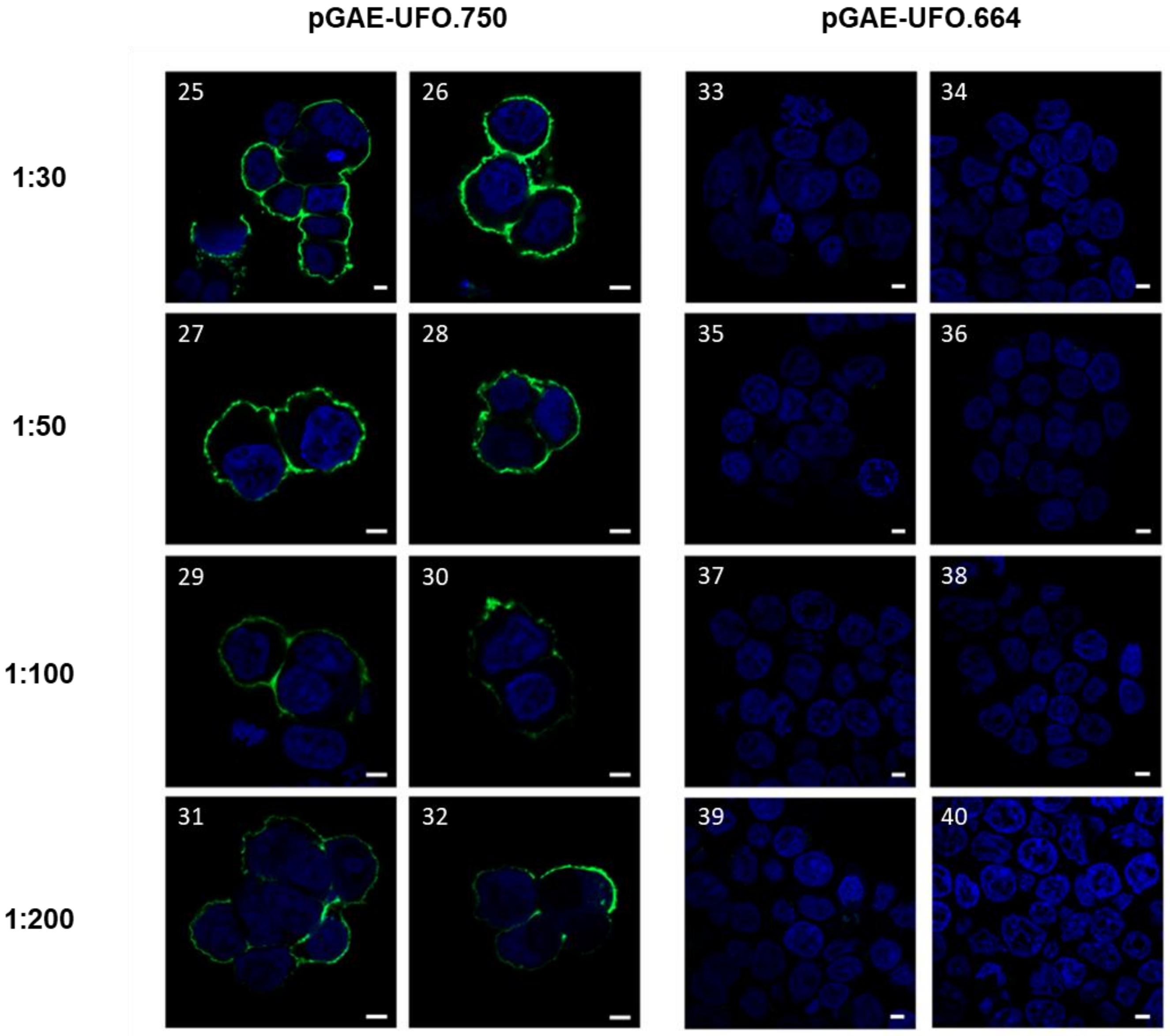

Supplementary Figure 2c

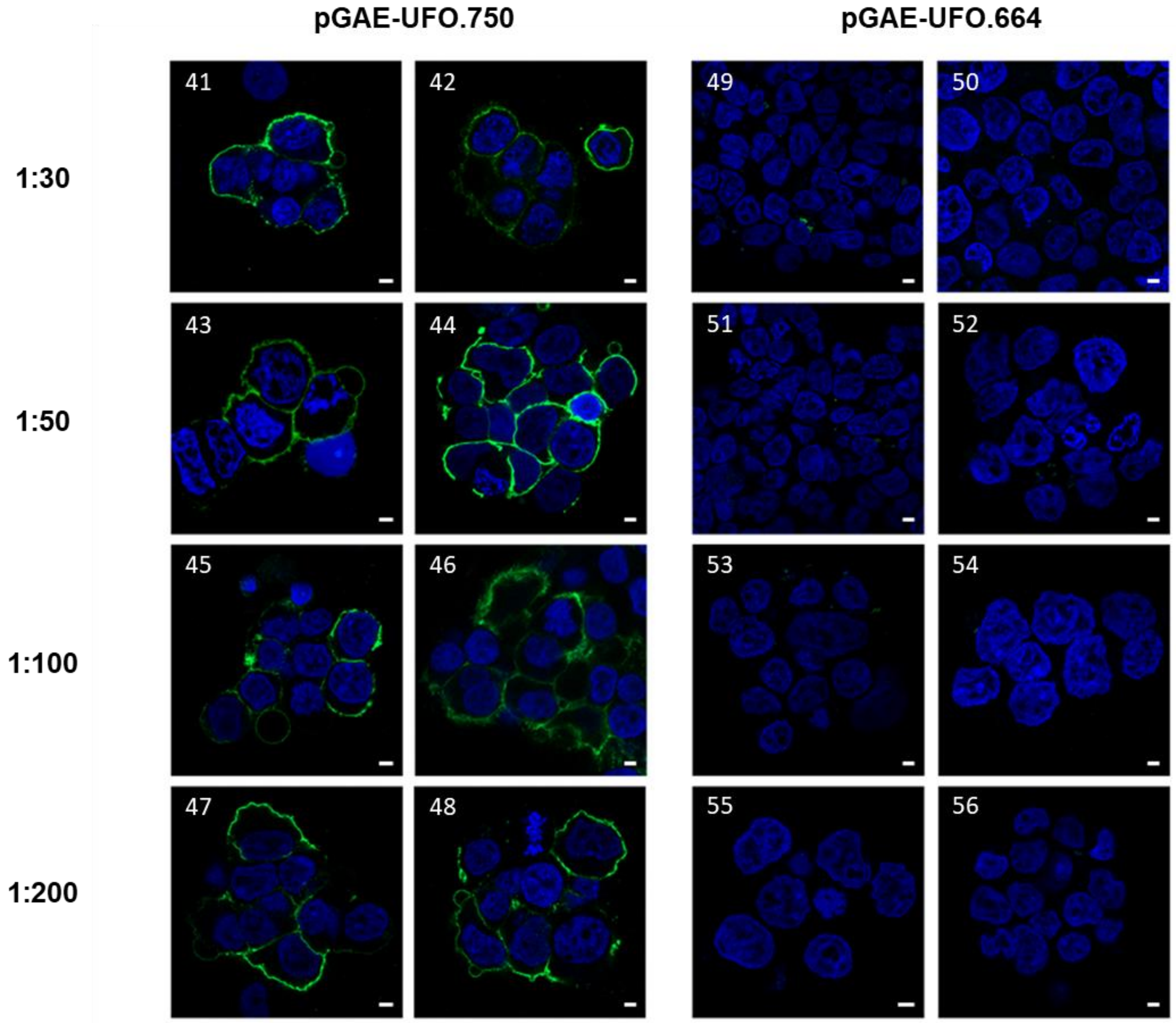

Supplementary Figure 2. **ConSOSL.UFO.664 and ConSOSL.UFO.750 expression from transfer vectors**. 293T Lenti-X cells were transfected with SIV-based transfer vectors pGAE-UFO.664 and pGAE-UFO.750 or with pConSgp160. Cells were membrane stained with scalar amount of anti-Env bNabs (**a**) 2G12 starting from 22,3 µg/sample (panels 1-24), (**b**) PGT145 starting from 8.84 µg/sample (panels 25-40) or (**c**) PGDM1400 starting from 4.42 µg/sample (panels 41-56) and analyzed by CSLM. Anti-human IgG Alexa Fluor 488 was used as secondary Ab. Nuclei were stained in blue by DAPI and scale bars is 5 µm. Two images are shown for each dose of bNAb. Images represent single central optical sections. Shown are results from one representative experiment.

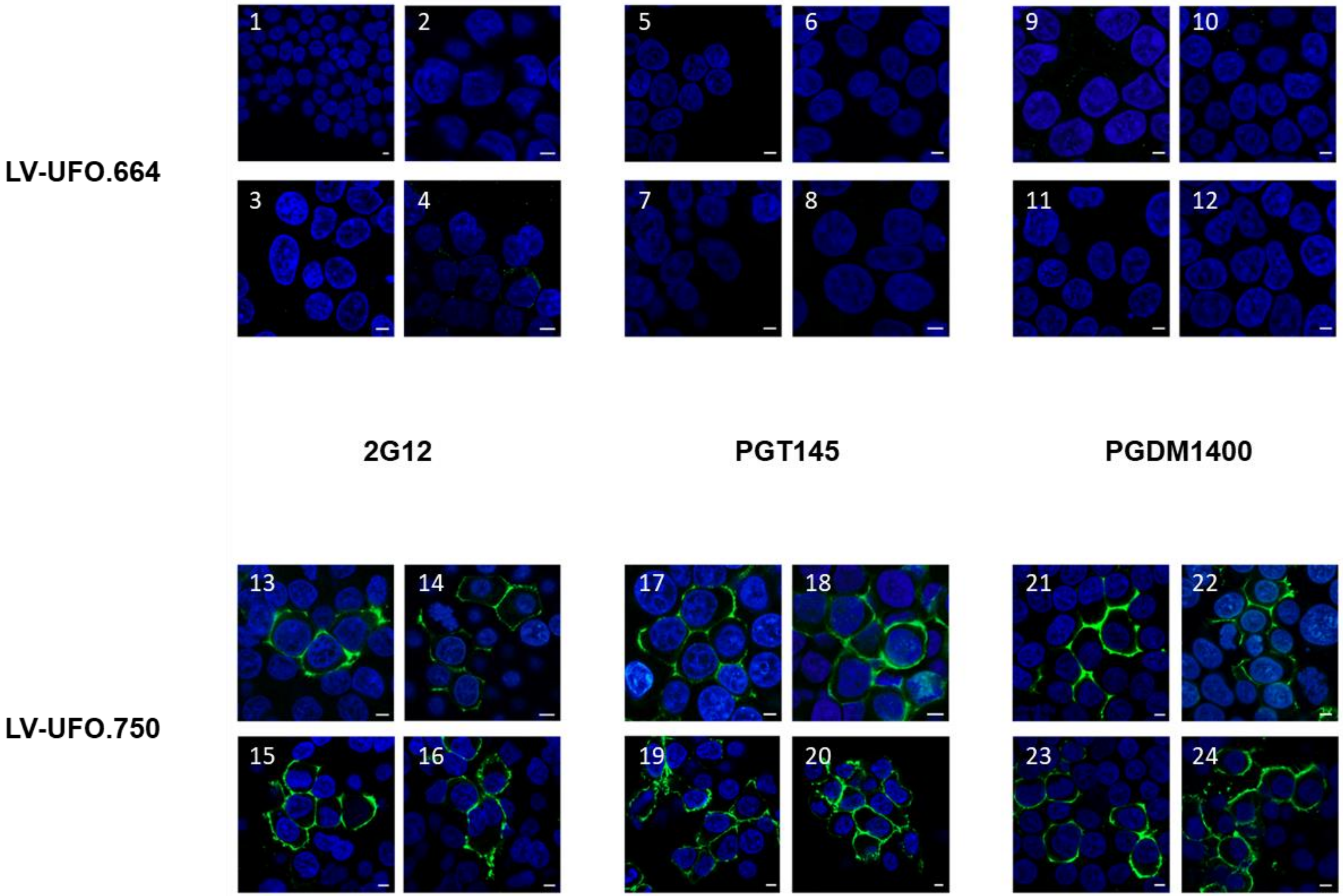

Supplementary Figure 3. **Expression of ConSOSL.UFO.664 and ConSOSL.UFO.750 from Lentiviral Vectors**. 293T Lenti-X cells were transduced with LV-UFO.664 or LV-UFO.750 and membrane stained with anti-Env nAbs 2G12 (13.1 µg/sample; panels 1-4, 13-16), PGT145 (5.2 µg/sample; panels 5-8, 17-20) and PGDM1400 (2.6 µg/sample; panels 9-12, 21-24). Anti-human IgG Alexa Fluor 488 was used as secondary Ab. Nuclei were stained in blue with DAPI and scale bars is 5 µm. Four images representing single central optical sections are shown for each bNAb staining. Shown are results from two representative (two panels for each) of n = 3 experiments.

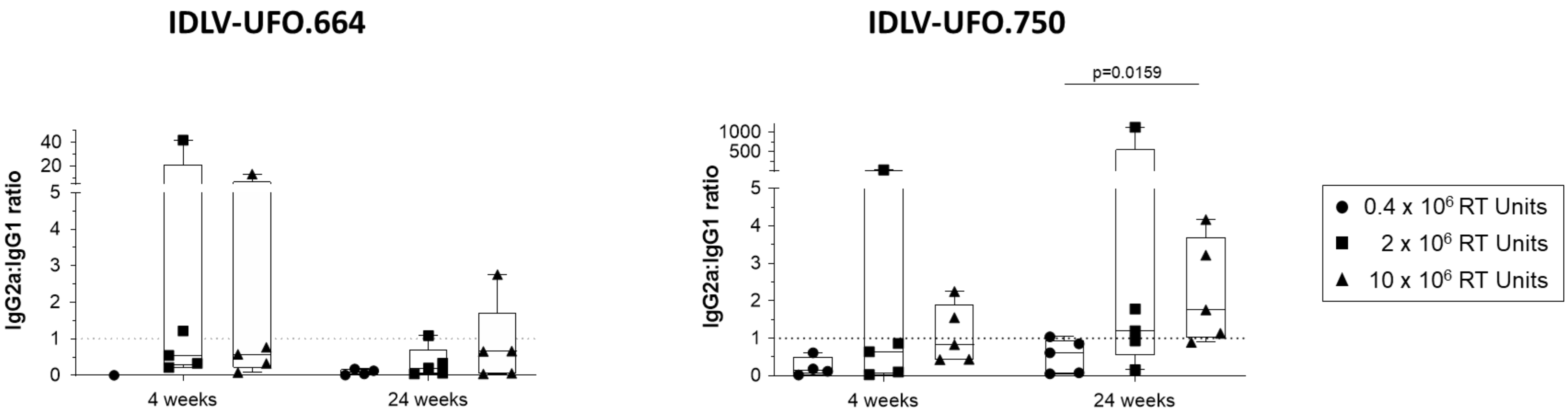

Supplementary Figure 4. **IDLV-UFO.750 induced a more pronounced Th1 response than IDLV-UFO.664 in immunized mice**. Sera from BALB/c mice vaccinated with escalating doses of IDLV-UFO.664 (left panel) or IDLV-UFO.750 (right panel) were analyzed by ELISA for ConSOSL.UFO-specific IgG1 and IgG2a concentration at weeks 4 and 24 from immunization. Results are expressed as IgG2a/IgG1 ratio and shown as box and whiskers. Each symbol represents a single animal (n=5 per group). Four out of 5 mice immunized with 0,4x106 IDLV-UFO.664 at week 4 and one out of 5 at week 24 were excluded from ratio analysis since both IgG2a and IgG1 were below 500 ng/mL. *p<0.05, Mann-Whitney test.

Black boxes cover samples not included in the manuscript

Figure 2a from the manuscript

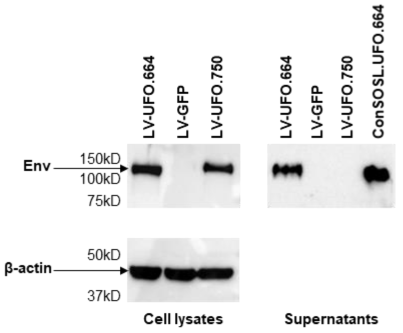

Chemiluminescence detection

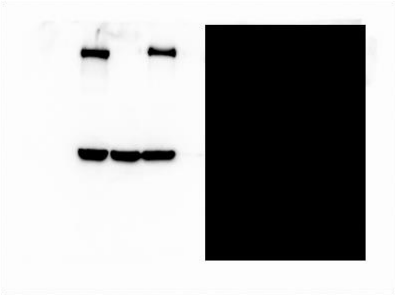

Marker and filter under visible light

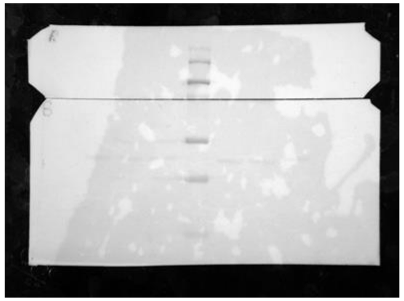

Merging of the two images showing position of marker and bands

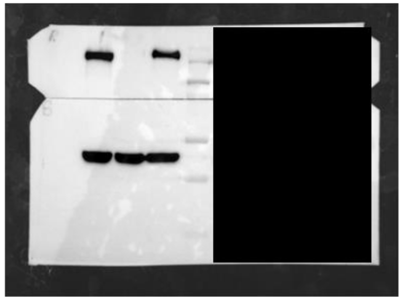

Chemiluminescence detection

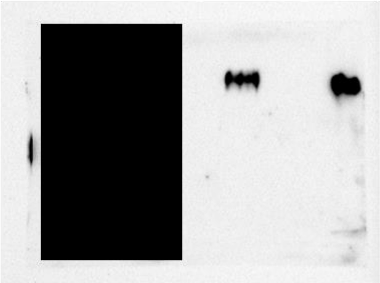

Marker and filter under visible light

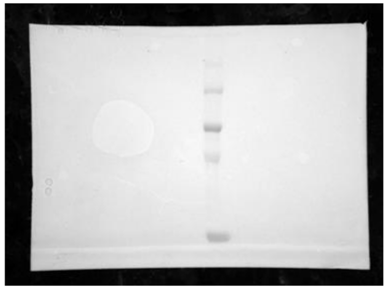

Merging of the two images showing position of marker and bands

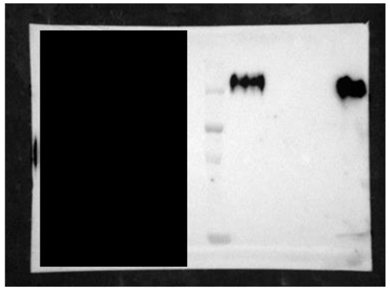

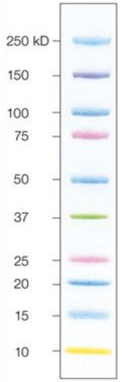

The Molecular Weight size marker is the Precision Plus Protein™ Kaleidoscope™ Prestained Protein Standards from BioRad
https://www.bio-rad.com/it-it/sku/1610375-precision-plus-protein-kaleidoscope-prestained-protein-standards?ID=1610375

Supplementary Figure 5. Figure 2a from the manuscript and scans of the original Western Blots.

Figure 3a and scans of the original Western Blot

Black boxes cover samples not included in the manuscript

Figure 3a from the manuscript

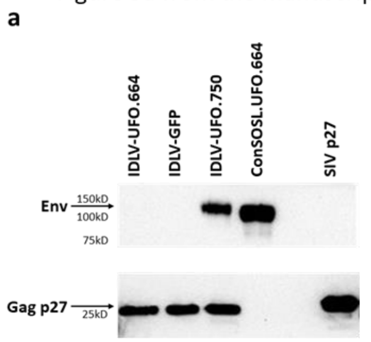

Chemiluminescence detection

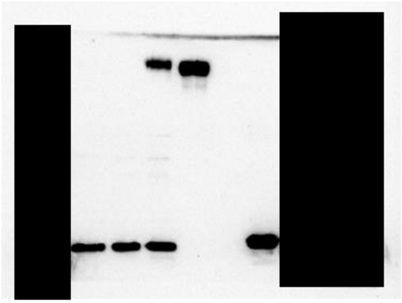

Marker and filter under visible light

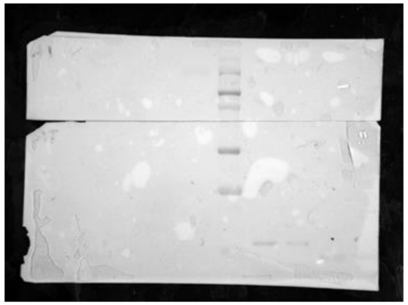

Merging of the two images showing position of marker and bands

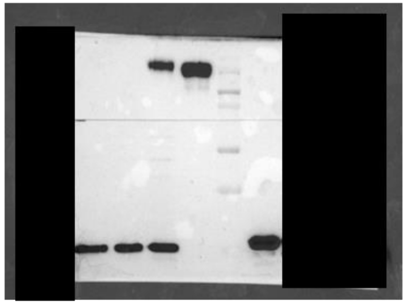

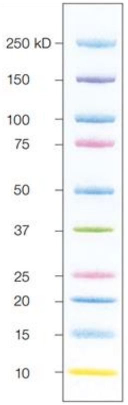

The Molecular Weight size marker is the Precision Plus Protein™ Kaleidoscope™ Prestained Protein Standards from BioRad
https://www.bio-rad.com/it-it/sku/1610375-precision-plus-protein-kaleidoscope-prestained-protein-standards?ID=1610375

Supplementary Figure 6. Figure 3a from the manuscript and scans of the original Western Blots.

Figure 3b and scans of the original Western Blot

Black boxes cover samples not included in the manuscript

Figure 3b from the manuscript

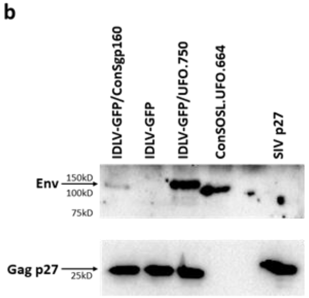

Chemiluminescence detection
Exposure: 155 sec for upper portion of the filter

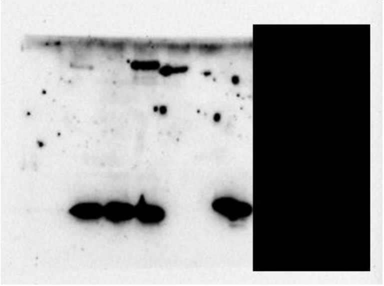

Chemiluminescence detection
Exposure: 55 sec for lower portion of the filter

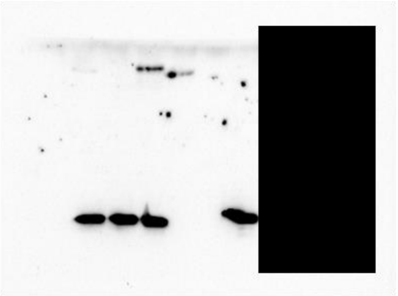

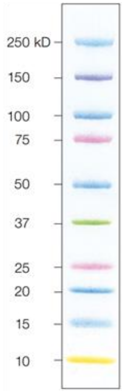

Marker and filter under visible light

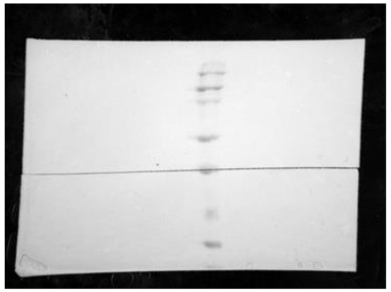

Merging of the two images showing position of marker and bands. Exposure: 155 sec

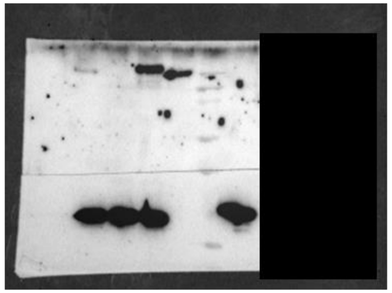

Merging of the two images showing position of marker and bands. Exposure: 55 sec

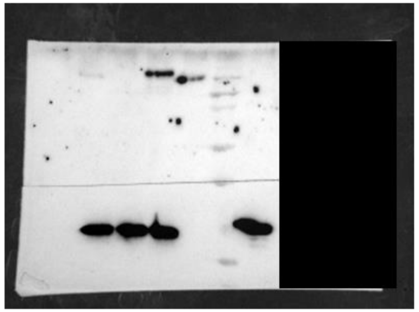

The Molecular Weight size marker is the Precision Plus Protein™ Kaleidoscope™ Prestained Protein Standards from BioRad
https://www.bio-rad.com/it-it/sku/1610375-precision-plus-protein-kaleidoscope-prestained-protein-standards?ID=1610375

Supplementary Figure 7. Figure 3b from the manuscript and scans of the original Western Blots.

Supplementary Table 1. Anti-ConSOSL.UFO.664 IgG in mucosal secretions from immunized monkeys.

	Animal ID	AS304		AT777		AU018		AU955		AU989	
Vaccine regimen	Week	Saliva	Rectal	Saliva	Rectal	Saliva	Rectal	Saliva	Rectal	Saliva	Rectal
IDLV-UFO750	0	0,02	0	0,01	0	0	0	0	0	0	0
	2	0,11	0	0,09	0,04	0.01	0	0	0	0	0
	6	0	0	0	0	0	0	0	0	0	0
	10	0	0	0	0	0	0	0	0	0	0
	14	0	0	0	0	0	0	0	0	0	0
	18	0	0	0,03	0	0	0	0	0	0	0
	22	0	0	0	0	0,13	0	0	0	0	0
	27	0	0	0	0	0	0	0	0	0	0,01
	33	0	0	0	0	0	0	0	0	0,13	0,02
IDLV-UFO750	37	0	0	0	0	0	0	0	0	0	0
	39	0,19	0	0,32	0	0	0	0,75	0	0,68	0,09
	43	0,17	0	0,27	0	0	0	0,16	0	0,43	0,03
	47	0	0	0,09	0	0	0	0	0,06	0	0,02
	52	0	0	0	0	0	0	0	0	0	0
	57	0	0	0	0	0	0	0	0	0	0
ConM SOSIP.v7 + MPLA	62	0	0	0	0	0	0	0	0	0,08	0
	64	0,17	0,02	0,16	0	0	0	0,98	0	0,34	0,01
	69	0,07	0	0,04	0	0	0	0	0,02	0	0,06
ConM SOSIP.v7 + MPLA	74	0	0	0	0	0	0	0,05	0,24	0	0
	76	0,15	0	0,18	0	0	0	1,43	0	0,38	0,05
	80	0,05	0	0,02	0	0	0	0,21	0	0,10	0
	85	0	0	0	0	0	0	0	0	0	0
	89	0	0	0,07	0	0	0	0	0	0	0
	94	0	0	0	0	0	0	0	0	0	0

Saliva and rectal swabs from immunized monkeys were collected at the indicated time points and analyzed by capture ELISA for the presence of anti-ConSOSL.UFO specific Abs. Results are expressed as the % of specific anti-ConSOSL.UFO.664 IgG compared to total IgG.

CTR- (untransduced Lenti-X cells)

Lenti-X cells transduced with LV-GFP

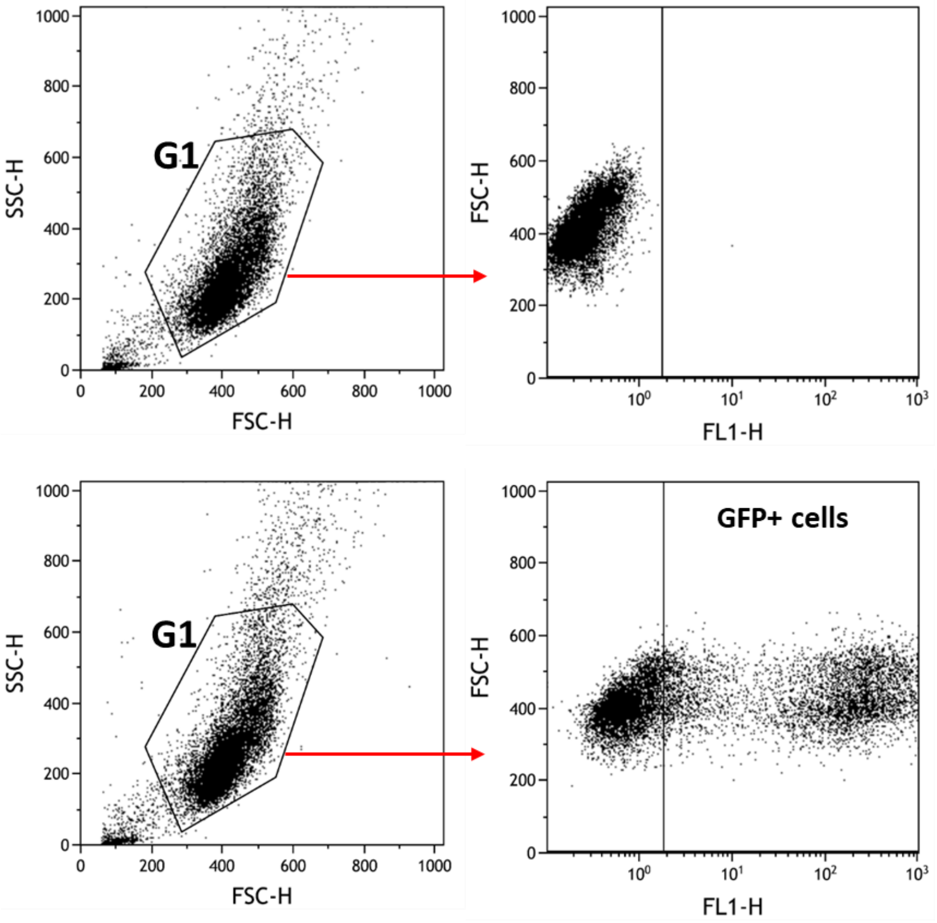

Gating Strategy. Forward (FSC) and side scatter (SSC) gating (G1) were used to identify the cells of interest, while removing debris. Lenti-X cells transduced with LV-GFP were gated and analysed for GFP expression. Untransduced Lenti-X cells was used as negative control (CTR-, upper panels) to set the quadrant and quantify the percentage of GFP-expressing cells (bottom panels).
