# Supplementary material for: Persistent immunogenicity of integrase defective lentiviral vectors delivering membrane-tethered native-like HIV-1 envelope trimers
Source: NPJ Vaccines. 2022 Apr 21;7:44. doi: 10.1038/s41541-022-00465-1 (PMC9023570; doi:10.1038/s41541-022-00465-1)
Supplement: Supplementary file 2 — REPORTING SUMMARY [file 41541_2022_465_MOESM2_ESM.pdf]

nature portfolio

Corresponding author(s): Andrea Cara

Last updated by author(s): Jan 20, 2022

Reporting Summary

Nature Portfolio wishes to improve the reproducibility of the work that we publish. This form provides structure for consistency and transparency in reporting. For further information on Nature Portfolio policies, see our Editorial Policies and the Editorial Policy Checklist.

Statistics

For all statistical analyses, confirm that the following items are present in the figure legend, table legend, main text, or Methods section.

n/a	Confirmed	
☐	☒	The exact sample size (*n*) for each experimental group/condition, given as a discrete number and unit of measurement
☐	☒	A statement on whether measurements were taken from distinct samples or whether the same sample was measured repeatedly
☐	☒	The statistical test(s) used AND whether they are one- or two-sided<br>*Only common tests should be described solely by name; describe more complex techniques in the Methods section.*
☒	☐	A description of all covariates tested
☐	☒	A description of any assumptions or corrections, such as tests of normality and adjustment for multiple comparisons
☐	☒	A full description of the statistical parameters including central tendency (e.g. means) or other basic estimates (e.g. regression coefficient) AND variation (e.g. standard deviation) or associated estimates of uncertainty (e.g. confidence intervals)
☐	☒	For null hypothesis testing, the test statistic (e.g. *F*, *t*, *r*) with confidence intervals, effect sizes, degrees of freedom and *P* value noted<br>*Give P values as exact values whenever suitable.*
☒	☐	For Bayesian analysis, information on the choice of priors and Markov chain Monte Carlo settings
☐	☒	For hierarchical and complex designs, identification of the appropriate level for tests and full reporting of outcomes
☐	☒	Estimates of effect sizes (e.g. Cohen's *d*, Pearson's *r*), indicating how they were calculated

Our web collection on statistics for biologists contains articles on many of the points above.

Software and code

Policy information about availability of computer code

Data collection	N/A
Data analysis	Zen Blue edition 3.3<br>Adobe Photoshop CS5<br>Kaluza Analysis Software<br>Softmax<br>Stata 16.1

For manuscripts utilizing custom algorithms or software that are central to the research but not yet described in published literature, software must be made available to editors and reviewers. We strongly encourage code deposition in a community repository (e.g. GitHub). See the Nature Portfolio guidelines for submitting code & software for further information.

Data

Policy information about availability of data

All manuscripts must include a data availability statement. This statement should provide the following information, where applicable:

- Accession codes, unique identifiers, or web links for publicly available datasets
- A description of any restrictions on data availability
- For clinical datasets or third party data, please ensure that the statement adheres to our policy

The data that support the findings of this study are available from the corresponding author upon reasonable request

March 2021

Field-specific reporting

Please select the one below that is the best fit for your research. If you are not sure, read the appropriate sections before making your selection.

☒ Life sciences ☐ Behavioural & social sciences ☐ Ecological, evolutionary & environmental sciences

For a reference copy of the document with all sections, see nature.com/documents/nr-reporting-summary-flat.pdf

Life sciences study design

All studies must disclose on these points even when the disclosure is negative.

Sample size	The number of NHP in the study (n=5) was pre-determined by funding availability. Given that all the animals responded to the vaccination, the sample size is sufficient to support the claimed results. The number of mice in the study (n=5 per group x 3 groups x 2 IDLV vaccines) was the minimum number of animals necessary to obtain statistically significant results. Given that all the animals responded to the vaccination, the sample size is sufficient to support the claimed results.
Data exclusions	No data were excluded
Replication	All attempts at replication were successful
Randomization	Animals were randomized among the different groups
Blinding	Personnel performing analysis of immune responses, luciferase activity tests, CLSM assay, were blinded to the different treatment groups

Reporting for specific materials, systems and methods

We require information from authors about some types of materials, experimental systems and methods used in many studies. Here, indicate whether each material, system or method listed is relevant to your study. If you are not sure if a list item applies to your research, read the appropriate section before selecting a response.

Materials & experimental systems

n/a	Involved in the study	
☐	☒	Antibodies
☐	☒	Eukaryotic cell lines
☒	☐	Palaeontology and archaeology
☐	☒	Animals and other organisms
☒	☐	Human research participants
☒	☐	Clinical data
☒	☐	Dual use research of concern

Methods

n/a	Involved in the study	
☒	☐	ChIP-seq
☐	☒	Flow cytometry
☒	☐	MRI-based neuroimaging

Antibodies

Antibodies used	Details provided in the manuscript
Validation	Details provided in the manuscript

Eukaryotic cell lines

Policy information about cell lines

Cell line source(s)	The human epithelium kidney 293T Lenti-X cell line used to produce IDLV particles, was obtained from Clontech Laboratories, Mountain View, CA. The VERO E6 cell line was obtained by ATCC (CRL-1586)
Authentication	Morphology was checked weekly by microscopy. Analysis of growth curves were performed routinely. Cells were cultured for no more than 20 passages.
Mycoplasma contamination	All cell line tested negative for mycoplasma contamination
Commonly misidentified lines (See ICLAC register)	No commonly misidentified cell lines were used

Animals and other organisms

Policy information about studies involving animals; ARRIVE guidelines recommended for reporting animal research

Laboratory animals	Six- to eight-week-old female BALB/c mice<br>Adult male cynomolgus monkeys (Macaca fascicularis: range 11.5-12.1 years old at the time of first immunization)
Wild animals	NA
Field-collected samples	NA
Ethics oversight	Mice were housed under specific pathogen-free conditions in the animal facility of the Istituto Superiore di Sanità (ISS, Rome, Italy). All animal procedures have been performed in accordance with European Union guidelines and Italian legislation for animal care. All animal studies were authorized by the Italian Ministry of Healthy and reviewed by the Service for Animal Welfare at ISS (Authorization n. 314/2015-PR of 30/04/2015)<br>Cynomolgus monkeys were housed under specific pathogen-free conditions in the animal facility of the Istituto Superiore di Sanità (ISS, Rome, Italy) according to the European Union guidelines and Italian legislation for non-human primate care. All animal studies were authorized by the Italian Ministry of Healthy and reviewed by the Service for Animal Welfare at ISS (Authorization n. 305/2016-PR of 24/03/2016).

Note that full information on the approval of the study protocol must also be provided in the manuscript.

Flow Cytometry

Plots

Confirm that:

- [x] The axis labels state the marker and fluorochrome used (e.g. CD4-FITC).
- [x] The axis scales are clearly visible. Include numbers along axes only for bottom left plot of group (a 'group' is an analysis of identical markers).
- [ ] All plots are contour plots with outliers or pseudocolor plots.
- [x] A numerical value for number of cells or percentage (with statistics) is provided.

Methodology

Sample preparation	293T Lenti-X human embryonic kidney cell line (Clontech, Mountain View, CA, USA) were transduced with LV-GFP pseudotyped with VSV.G serotypes
Instrument	FACScalibur, Becton Dikinson
Software	Kaluza software
Cell population abundance	NA
Gating strategy	Forward (FSC) and side scatter (SSC) gating (G1) was used to identify the cells of interest, while removing debris. Lenti-X cells transduced with LV-GFP were gated and analysed for GFP expression. Untransduced Lenti-X cells was used as negative control to set the quadrant and quantify the percentage of GFP-expressing cells.

- [x] Tick this box to confirm that a figure exemplifying the gating strategy is provided in the Supplementary Information.
